# Surgical Approaches to Perineal Reconstruction: A Narrative Review

**DOI:** 10.7759/cureus.116099

**Published:** 2026-09-11

**Authors:** David A Febre-Alemañy, Michael J Franco

**Affiliations:** 1 Surgery, San Juan Bautista School of Medicine, Caguas, PRI; 2 Plastic and Reconstructive Surgery, Cooper University Hospital, Camden, USA

**Keywords:** abdominoperineal resection, fournier gangrene, gluteal flap, pelvic exenteration, perforator flap, perineal reconstruction, vertical rectus abdominis myocutaneous flap, vulvar reconstruction

## Abstract

The reconstruction of the perineum remains one of the most demanding fields in reconstructive surgery. The region combines a contaminated operative field, a high prevalence of prior radiotherapy, complex three-dimensional dead space, and functional structures (the anus, urethra, and vagina) whose preservation or restoration directly determines the quality of life. The principal indications for perineal reconstruction are oncologic resection, most commonly abdominoperineal resection and pelvic exenteration for anorectal and gynecologic malignancy, radical vulvectomy, and the soft tissue destruction produced by Fournier gangrene. This narrative review traces the historical evolution of perineal reconstruction from the era of open wound packing to modern perforator flap surgery, summarizes the anatomic and physiologic principles that underpin flap-based repair, and appraises the current evidence for the main reconstructive options, including primary closure, biologic mesh, omental flaps, the vertical rectus abdominis myocutaneous (VRAM) flap, gluteal and thigh-based flaps, and fasciocutaneous perforator flaps such as the lotus petal flap. Comparative data from systematic reviews, meta-analyses, and the recent BIOPEX-2 and Nordic Extended Abdominoperineal Excision (NEAPE) randomized trials are discussed, together with functional, sexual, and quality of life outcomes and reconstructive strategy in Fournier gangrene. A defect-oriented algorithmic approach, tailored to the resection performed, the radiation field, patient factors, and donor site availability, currently offers the most rational framework for choosing among reconstructive options.

## Introduction and background

Perineal defects arise principally from oncologic resection (abdominoperineal resection and pelvic exenteration for rectal and anal cancer and radical vulvectomy and exenterative surgery for gynecologic malignancy); from necrotizing soft tissue infection, chiefly Fournier gangrene; and less commonly from trauma, hidradenitis suppurativa, inflammatory bowel disease, and congenital conditions [1,2]. The perineum is an unforgiving site for wound healing: The field is contaminated or clean-contaminated by definition, the wound is dependent and exposed to pressure and shear with sitting, the pelvic cavity left behind by resection cannot collapse, and the patient population carries a heavy burden of prior radiotherapy, obesity, diabetes, smoking, malnutrition, and immunosuppression [2,3]. The region is also functionally dense, so reconstruction must respect or restore the anus, urethra, and vagina; allow pain-free sitting; and preserve continence where sphincters remain [2]. This review summarizes the historical development, principles, techniques, comparative evidence, and functional outcomes of perineal reconstruction, with the aim of providing a defect-oriented framework for reconstructive decision-making.

Historical background

The history of perineal reconstruction is inseparable from that of radical pelvic surgery. When Miles described the abdominoperineal excision of the rectum in 1908, he established the operation that would define low rectal cancer surgery for the following century and, with it, a wound that surgeons would struggle to close for just as long [4]. For decades, the perineal defect was managed with loose approximation, gauze packing, and healing by secondary intention, and chronic sinuses and months of dressing changes were accepted as the price of cure. Even today, the perineal wound remains the Achilles' heel of the operation, with wound problems reported in up to 57% of patients [3].

Two mid-20th-century developments changed the conceptual landscape. The first was the recognition that well-vascularized muscle could be transposed into compromised wounds. The musculocutaneous flap principle, articulated by Orticochea in 1972 as an alternative to staged, delayed flaps, made single-stage transfer of composite tissue a practical reality [5]. It reached the perineum in 1976, when McCraw et al. reported vaginal reconstruction with gracilis myocutaneous flaps after pelvic exenteration [6]. During the 1980s, the rectus abdominis myocutaneous flap, raised on the deep inferior epigastric pedicle, was adapted to pelvic and perineal defects, and its vertical skin paddle version, the vertical rectus abdominis myocutaneous (VRAM) flap, progressively displaced the gracilis as the workhorse for large postexenterative defects because of its reliable pedicle, generous bulk, and long arc of rotation [7,8].

The second development was anatomic. Koshima and Soeda's inferior epigastric artery skin flap without rectus muscle, reported in 1989, launched the perforator flap era and its promise of equivalent soft tissue transfer with reduced donor morbidity [9]. The 1990s saw the progressive refinement of perforator flap techniques, with Koshima et al. [10] describing the free thin paraumbilical perforator flap, the direct precursor of the deep inferior epigastric perforator (DIEP) flap, in 1992, and Allen and Treece [11] popularizing the DIEP flap for breast reconstruction in 1994. These principles were progressively applied to perineal defects, whose rich anastomotic blood supply from the internal and external pudendal systems proved fertile ground for the concept. Wee and Joseph described the neurovascular pudendal thigh flap for vaginal reconstruction in 1989 [12], and the lotus petal flap described by Yii and Niranjan in 1996, a fasciocutaneous petal raised on perforators around the perineum and gluteal fold, represented a significant milestone in local perforator-based reconstruction of the vulvoperineal region [13]. By 2010, reviews were explicitly framing perforator flaps as a new option in perineal reconstruction [14].

Meanwhile, oncologic surgery itself enlarged the reconstructive problem. Total mesorectal excision improved oncologic control but exposed the vulnerability of the conventional abdominoperineal specimen, wasted at the level of the levators, to margin involvement and perforation. In response, Holm et al. described in 2007 the extended, cylindrical abdominoperineal excision performed prone, resecting the levator plate en bloc and reconstructing the pelvic floor with a gluteus maximus flap [15]. The extralevator approach and the widespread use of neoadjuvant radiotherapy, which more than doubles the odds of perineal wound problems, substantially increased the size and hostility of the defect [3]. Reconstruction thus moved from an occasional salvage maneuver to an anticipated component of the primary operation, and the last two decades have brought randomized trials of biologic mesh, gluteal flaps, and primary closure [16-18]. The century since Miles [4] thus describes a coherent arc, from wounds left open, to muscle brought in from a distance, to refined local tissue raised on named perforators, and finally to randomized evidence and algorithmic, defect-oriented planning [1,2].

Methods

This narrative review was conducted to synthesize the current evidence on the reconstruction of perineal defects across oncologic and nononcologic indications. Although narrative reviews do not require the systematic methodology of a formal systematic review, we followed a structured approach to literature identification and selection to enhance transparency and reproducibility, consistent with the Scale for the Assessment of Narrative Review Articles (SANRA) criteria.

A comprehensive literature search of PubMed/Medical Literature Analysis and Retrieval System Online (MEDLINE), Embase, and the Cochrane Library was performed from database inception through August 2026. Search terms combined free text and Medical Subject Headings related to the anatomic region and reconstructive techniques, including "perineal reconstruction," "perineal defect," "vulvoperineal reconstruction," "vulvar reconstruction," "abdominoperineal resection," "extralevator abdominoperineal excision," "pelvic exenteration," "Fournier gangrene," and "perineal wound closure," and terms for individual techniques such as "vertical rectus abdominis myocutaneous flap," "gracilis flap," "anterolateral thigh flap," "gluteal fold flap," "lotus petal flap," "perforator flap," "biological mesh," and "primary closure." Boolean operators were used to combine terms, and searches were limited to studies published in English. Reference lists of included articles and prior reviews were hand-searched to identify additional relevant publications not captured by the database search.

Articles were eligible for inclusion if they reported on the surgical reconstruction of perineal, vulvar, or vulvoperineal defects in adult patients, including randomized controlled trials, systematic reviews and meta-analyses, prospective and retrospective cohort studies, comparative studies, and large case series. Case reports were considered only when they described novel techniques not otherwise represented in the literature. Studies addressing exclusively pediatric populations, gender-affirming genital surgery, and isolated urethral or penile reconstruction were excluded, as were conference abstracts without full text, editorials, and letters. Given the narrative design, no formal risk of bias assessment or quantitative pooling was performed. Extracted information included study design, sample size, indication for reconstruction, reconstructive technique, wound and donor site complications, and functional and quality of life outcomes. Findings were synthesized thematically into the following domains: reconstructive principles and algorithmic approaches, closure strategies after abdominoperineal resection and extralevator abdominoperineal excision, flap options and comparative outcomes, vulvoperineal and perforator-based reconstruction, nononcologic indications, and functional and patient-reported outcomes.

## Review

Search strategy and summary of the evidence base

This narrative review was prepared in accordance with the general principles of the Scale for the Assessment of Narrative Review Articles (SANRA); PRISMA reporting does not apply to a narrative synthesis. A targeted search of PubMed/MEDLINE was performed for English-language articles published from January 1980 through August 2026 using combinations of the terms perineal reconstruction, perineal wound, perineal closure, vulvoperineal reconstruction, vulvar reconstruction, Fournier gangrene, abdominoperineal resection, pelvic exenteration, flap, perforator flap, mesh, and primary closure, supplemented by the hand searching of the reference lists of retrieved reviews and of the historical literature. The search returned 1,010 records. After the removal of duplicates and title and abstract screening for relevance to reconstructive technique, comparative outcomes, or functional and quality of life outcomes, 36 contemporary full-text articles were retrieved and assessed, all of which were included, and eight landmark historical primary sources identified from reference lists were added, yielding 44 references. Priority was given to randomized trials, systematic reviews and meta-analyses, and the largest or most recent cohort studies for each reconstructive technique.

The resulting evidence base comprises three randomized clinical trial reports (the BIOPEX-2 primary outcome and cost-effectiveness analyses and the Nordic Extended Abdominoperineal Excision {NEAPE} trial) [16-18]; seven systematic reviews or meta-analyses [3,19-24]; five narrative reviews and educational articles [1,2,7,14,25]; 21 original cohort studies, case series, or cross-sectional studies [8,13,15,26-43]; and eight historical primary sources [4-6,9-12,44]. The 21 original clinical studies together report on approximately 1,800 patients (the BIOPEX-2 cohort counted once), of whom 546 belong to a single institutional VRAM series [27] and 334 to the multicenter PelvEx gynecologic reconstruction cohort [38]. The included meta-analyses pool a further 1,141 patients comparing primary closure with VRAM closure after abdominoperineal resection [19], 1,840 patients undergoing perforator or non-perforator vulvoperineal reconstruction [22], and 576 reconstructions after Fournier gangrene [24]. Ten of the 36 contemporary sources were published in 2024 or later, reflecting the recent acceleration of comparative research in this field.

The perineal defect: Etiology and rationale for reconstruction

Several features conspire to make perineal wounds uniquely difficult. The defect frequently follows neoadjuvant chemoradiotherapy, which impairs the healing of the wound edges and of the pelvic cavity; in the meta-analysis by Musters et al., perineal wound problems occurred in 15.3% of patients after conventional abdominoperineal resection without radiotherapy but in 30.2% after radiotherapy, and radiotherapy independently increased wound problems (odds ratio: 2.22) [3]. The resection creates a rigid, noncollapsible pelvic dead space that fills with fluid and is prone to abscess formation and chronic sinus, a situation aggravated in the extralevator and exenterative variants and contributing to the so-called empty pelvis syndrome [19,27,28]. The dependent position of the wound, contamination, and patient comorbidity compound these problems [2].

The rationale for flap-based reconstruction follows directly from these features. Transposing well-vascularized, nonirradiated tissue obliterates dead space, imports blood supply into an ischemic field, provides skin to bridge irradiated wound edges, separates visceral suture lines, and permits the reconstruction of the vaginal wall or perineal skin as required [8,27,28]. The consistent message of the comparative literature, discussed below, is that these theoretical advantages translate into measurably fewer major perineal wound complications in irradiated and extended resections, at the cost of longer operations and donor site morbidity, so that the central question of modern practice is not whether vascularized tissue helps but which patients need it and which donor site serves them best [19,20,28].

Principles and goals of reconstruction

The goals of perineal reconstruction can be summarized as the obliteration of pelvic and perineal dead space with vascularized tissue, tension-free and durable skin closure, the maintenance or restoration of function (vaginal patency, an unobstructed anal or urethral orifice where preserved, and the capacity for comfortable sitting), the prevention of perineal herniation, and an acceptable donor site [1,2]. Westbom and Talbot emphasize that flap choice is constrained as much by what is available as by what is ideal: The abdominal donor site may be unavailable because of stomas, hernias, morbid obesity, or prior laparotomy, and the prior use of gracilis or pudendal territories may eliminate them from consideration [2]. Sinna et al. proposed a practical decision structure organized around the surgical approach itself: When laparotomy is already required, abdominally based flaps are preferred because they are raised in the same field and passed through the pelvis; when the perineal phase is performed prone, as in extralevator excision, gluteal and pudendal donor sites are recommended [14]. Multidisciplinary planning among the resecting surgeon, plastic surgeon, radiologist, and anesthetist before the operation, rather than intraoperative improvisation, is repeatedly identified as a determinant of success [1,14].

Reconstructive options

Primary Closure and Biologic Mesh

Primary layered closure remains appropriate for limited defects in nonirradiated fields with preserved levators. Its limitations in the irradiated, extended resection are the central lesson of the comparative literature: in the meta-analysis of Temperley et al. covering 10 studies and 1,141 patients, perineal wound complications occurred in 38.2% of primary closure patients versus 32.8% after VRAM closure, a statistically significant advantage for the flap (odds ratio, 1.61; p = 0.03) [19]. The biologic mesh closure of the pelvic floor emerged as an intermediate option that avoids a donor site; pooled data suggested encouragingly low perineal wound problem rates in selected series [3]. Randomized evidence has, however, tempered enthusiasm. In the Nordic NEAPE trial, which randomized patients undergoing extended abdominoperineal excision to an acellular porcine collagen implant or a unilateral gluteus maximus myocutaneous flap, the implant failed to demonstrate non-inferiority for physical function at six months (71% versus 60% underperforming on the timed-stands test), with the direction of effect favoring the flap [18].

Omental Flaps

The pedicled omental flap reaches the pelvis reliably, obliterates dead space, and adds no cutaneous donor site, and it is used both alone for moderate dead space and as an adjunct to skin or myocutaneous closure [1,28]. In the single-center longitudinal series of Gala et al., omental flaps formed a small but useful part of the reconstructive mix, alongside VRAM and gracilis flaps [28]. Its principal limitations are variable volume, dependence on an intact and unoperated omentum, and the need for transabdominal harvest.

Abdominally Based Flaps: The VRAM Flap and Its Variants

The vertical rectus abdominis myocutaneous flap is widely regarded as the reference standard for large perineal and pelvic defects, particularly after irradiated abdominoperineal resection and pelvic exenteration with or without vaginal resection [8,20,27]. Raised on the deep inferior epigastric pedicle with a vertical skin paddle, it delivers robust muscle bulk for the pelvic cavity and a reliable skin island for the perineum or posterior vagina. In the largest reported single-institution experience, 546 consecutive VRAM pelvic reconstructions over 20 years, recipient site surgical occurrences developed in 38% and donor site occurrences in 17% of patients, with hernia in 9.9% and bulge in 6.4%; tobacco use and the use of an extended flap approximately doubled hernia risk, while mesh reinforcement and component separation were not independently protective [27]. Comparative data support its primacy among flaps for the largest defects: in the series of Nelson and Butler, immediate VRAM reconstruction produced significantly fewer major complications than thigh-based flaps (15% versus 42%), with lower rates of pelvic abscess and major dehiscence and without increased early abdominal wall morbidity [8]. The flap's liabilities are the laparotomy and donor defect it requires and its unavailability when stomas, prior surgery, or planned abdominal wall reconstruction preclude harvest [2]. Fistula-prone patients merit caution: in the longitudinal series of Gala et al., enterocutaneous fistula formation was significantly more frequent after VRAM than after gracilis reconstruction when adjusted for neoadjuvant chemoradiation [28]. Muscle-sparing and perforator variants (MS-VRAM and DIEP-based reconstruction) aim to preserve rectus function while retaining the pedicle's reach, and the DIEP flap has an established role in vulvar and vaginal reconstruction [14,29].

Gluteal Flaps

Gluteus maximus-based reconstruction is the natural partner of the prone, extralevator excision, since the donor site lies in the operative field and the abdomen is spared. Holm et al.'s original description paired the cylindrical excision with a gluteus maximus flap for the pelvic floor, reporting low rates of perforation, margin involvement, and perineal wound complications in the initial cohort [15]. Long-term function after unilateral gluteus maximus rotation flap reconstruction appears acceptable: in a cohort examined with a mean of 26 months after extended excision, balance and hip mobility were essentially normal (mean Berg balance score of 52.8 of 56), although sitting tolerance was reduced in a proportion of patients and only nine of 19 were entirely pain-free while seated [30]. The gluteal turnover flap, a deepithelialized local advancement that fills the perineal defect without a visible additional scar, was tested against primary closure in the multicenter BIOPEX-2 randomized trial: uncomplicated perineal wound healing at 30 days did not differ (55% versus 60%), so superiority was not shown [17]. The accompanying cost-effectiveness analysis nonetheless favored the flap, as discussed in detail below [16]. Gluteal fold and inferior gluteal artery perforator (IGAP) flaps extend the same territory on perforator principles, and gluteal transposition flaps have been used for combined perineal and vaginal reconstruction after abdominoperineal excision with partial vaginectomy, with major flap-specific complications in 9% in a two-center series [31].

Thigh-Based Flaps: Gracilis and Anterolateral Thigh (ALT)

The gracilis, the historical first mover in perineal reconstruction, remains valuable because its harvest spares the abdomen and its donor morbidity is modest [6]. In a proportional meta-analysis comparing VRAM to gracilis flaps for vulvoperineal reconstruction after oncologic resection, pooled donor site complications were substantially higher for VRAM (57.6%) than for gracilis (16.0%), although the skin paddle of the gracilis is less reliable and partial necrosis remains its characteristic weakness [20]. Contemporary series position the gracilis as a first-line alternative to VRAM in appropriately selected patients, particularly those predisposed to fistula or in whom the abdominal wall must be preserved [28]. The pedicled anterolateral thigh (ALT) flap, with or without vastus lateralis, brings a large, well-vascularized skin paddle and muscle bulk to the perineum without violating the abdomen. In a consecutive series of 29 composite ALT vastus lateralis flaps for postoncologic abdominoperineal defects, flap survival was 100%, and initial defect size of 190 square centimeters or more was the principal predictor of complications [32]. In vulvar oncology, ALT-based reconstruction in its unilateral, combined, fenestrated, and split designs has provided reliable coverage of extensive defects with minimal donor morbidity [33].

Fasciocutaneous and Perforator Flaps of the Perineal Region

The lotus petal flap and its relatives (gluteal fold, internal and external pudendal artery perforator, and pudendal thigh flaps) exploit the perineum's densely anastomotic blood supply to provide thin, pliable, like-with-like tissue for vulvar, vaginal, and perineal resurfacing [12,13]. The largest single-center analysis of lotus petal reconstruction, 93 patients and 137 flaps, reported no complications in 30.1% of patients, minor (Clavien-Dindo I-II) complications in 51.7%, reintervention under general anesthesia in 18.3%, and donor site morbidity in 14% [34]. A systematic review comparing lotus petal to VRAM reconstruction in the vulvoperineal area found lower recipient site (26.0% versus 37.1%) and donor site (4.5% versus 17.8%) complication rates for the lotus petal flap and suggested that when both options are feasible, the lotus petal flap may be preferred [21]. Suprafascial harvest simplifies elevation and preserves the deep fascia [35], and defect topography-based algorithms combining lotus petal and DIEP flaps have been shown to cover essentially all acquired vulvar defects [29]. At the level of pooled evidence, a meta-analysis of 1,840 patients undergoing vulvoperineal reconstruction found comparable short-term complication rates between perforator and nonperforator flaps, with a tendency toward fewer complications and fewer functional deficits with perforator-based reconstruction [22]. The perforator toolkit continues to expand, as illustrated by the deep external pudendal artery perforator (DEPAP) flap recently described for perineal defects after Fournier gangrene [36].

Comparative evidence after abdominoperineal resection and pelvic exenteration

The comparative literature now permits some reasonably firm statements. First, in irradiated and extended resections, flap reconstruction reduces perineal wound morbidity relative to primary closure. The meta-analysis of Buscail et al. [23] found primary closure associated with increased total and major perineal wound complications compared with VRAM closure after abdominoperineal resection, a pattern echoed by Temperley et al. [19]. Notably, in the pelvic exenteration subgroup of the Buscail et al. analysis, this relationship inverted, a reminder that selection bias pervades the retrospective literature and that the largest flaps are applied to the largest, most hostile defects [23]. Second, the benefit of routine flap closure in less extended resections is not established. BIOPEX-2, the only masked randomized comparison of a flap technique against primary closure after abdominoperineal resection, showed no superiority of the gluteal turnover flap for uncomplicated 30-day healing [17]. Third, biologic mesh is not a proven substitute for vascularized tissue in the extended excision, as the NEAPE trial's failed non-inferiority demonstrates [18]. Fourth, among flaps, the VRAM remains the volume and reliability standard for the largest pelvic defects [8,27], while gracilis, ALT, gluteal, and perforator alternatives narrow the gap for intermediate defects with materially lower donor morbidity [20,21,32]. Overall complication rates after perineal reconstruction remain high in every series, commonly between 20% and 50% and exceeding 70% in some longitudinal cohorts when all wound events are counted, which underscores that reconstruction mitigates rather than eliminates the morbidity of these operations [7,28].

Cost-Effectiveness

Economic evidence in perineal reconstruction is sparse, and the BIOPEX-2 cost-effectiveness analysis is the only randomized economic evaluation of a flap technique in this setting [16]. Over a 12-month horizon, the mean total healthcare cost per patient was 53,397 euros (95% confidence interval: 46,645-60,149) after primary closure and 45,079 euros (42,201-47,957) after gluteal turnover flap closure, a mean saving of 8,318 euros (928-15,709; p = 0.027) in favor of the flap. The flap itself was slightly more expensive to perform, adding a mean of 35 minutes of operating time at an estimated 388 euros, but this was offset many times over by downstream savings: total outpatient facility costs of 53,387 versus 71,316 euros, mean specialized wound care costs of 720 versus 1,200 euros, mean home care costs of 2,504 versus 3,842 euros, and fewer reinterventions and readmissions, the most expensive of which was perineal hernia repair (approximately 16,264 euros per event) and surgery for ileus. The savings persisted when productivity losses were included using the friction cost method (8,553 euros; p = 0.046) and widened, although without reaching statistical significance, under the human capital method (21,450 euros).

Patients who developed perineal complications reported significantly lower EQ-5D and EQ-VAS scores over time than those who did not, although the quality of life did not differ significantly between the randomized groups as a whole [16]. Two features of this analysis deserve emphasis. First, the economic benefit was demonstrated even though the primary clinical end point of the parent trial was not met, indicating that a reduction in the severity and downstream burden of wound complications, rather than in their 30-day incidence, drove the savings [16,17]. Second, the gluteal turnover flap can be performed by a trained colorectal surgeon without a plastic surgery team and without a separate donor site, so its cost profile may not generalize to VRAM, gracilis, or ALT reconstruction, for which formal economic evaluations are lacking [16]. Indirect cost signals nonetheless point in the same direction for these flaps: in the longitudinal series of Gala et al., 45% of patients required a procedural intervention for a reconstructive complication [28], and the large VRAM series of Asaad et al. documented a 9.9% hernia rate that carries its own repair costs [27], underscoring that the economic case for any reconstruction rests on preventing expensive secondary events rather than on the cost of the index procedure. Cost-effectiveness analyses of biologic mesh against flap reconstruction and comparisons among flap classes remain priorities for future research.

Summary of Quantitative Findings

Taken together, the included studies support the following summary estimates. Perineal wound problems after primary closure occur in roughly 15% of nonirradiated and 30% of irradiated abdominoperineal resections, with radiotherapy conferring an odds ratio of 2.22 [3]. Flap closure with the VRAM reduces perineal complications from 38.2% to 32.8% relative to primary closure (odds ratio: 1.61) [19] but at the price of donor site complications in 17%, hernia in 9.9%, and bulge in 6.4% [27]. Donor site morbidity is roughly three to four times lower with the gracilis than with the VRAM (16.0% versus 57.6% pooled) [20], and recipient and donor site complication rates are lower with the lotus petal flap than with the VRAM (26.0% versus 37.1% and 4.5% versus 17.8%) [21]. Perforator and nonperforator flaps carry comparable overall complication rates of approximately 30% in vulvoperineal reconstruction [22]. In randomized comparisons, the gluteal turnover flap achieved uncomplicated 30-day healing in 55% of patients versus 60% with primary closure (not significant) while saving 8,318 euros per patient [16,17], and the collagen implant left 71% of patients underperforming on the timed-stands test versus 60% after gluteus maximus flap reconstruction [18]. In Fournier gangrene, more than three-quarters of reconstructions are accomplished with grafts, local random flaps, or secondary healing [24]; overall complication rates of 16%-27% are reported in defect-based series [39,40]; and gracilis interposition for perianal dead space shortens the interval to discharge from 12.7 to 5.1 days [41].

Vulvovaginal reconstruction

Vulvovaginal defects merit separate consideration because the reconstructive aim extends beyond wound closure to the restoration of body image, vaginal capacity, and sexual function, in a population that is often younger than the rectal cancer population [29,38]. Options range from local advancement and lotus petal flaps for superficial vulvar defects, through pudendal thigh and gluteal fold flaps for lateral and posterior defects, to ALT, DIEP, and VRAM flaps for radical and exenterative defects, with topography-based algorithms guiding selection [2,29,33]. Where the posterior vaginal wall is resected with the rectum, immediate reconstruction with gluteal transposition flaps or myocutaneous flaps restores the canal, although functional recovery is incomplete: In the series of Bolmstrand et al., only 45% of previously sexually active survivors resumed sexual activity, and 27% resumed vaginal intercourse, all of the latter having followed an active vaginal health program [31]. In the PelvEx Collaborative's multicenter analysis of gynecologic reconstruction in pelvic exenteration (334 patients), reconstruction lengthened operating time and hospital stay and increased minor perineal complications without increasing reintervention; procedure-specific major morbidity was 5.2% for neovaginal and 11.5% for flap reconstruction, two-thirds of neovaginal patients had no long-term morbidity, and neovaginal stenosis occurred in 7% [38]. The collaborative concluded that neovaginal reconstruction is safe in carefully selected patients, while primary closure does not itself increase perineal morbidity in this setting [38].

Reconstruction in Fournier gangrene

Jean Alfred Fournier, the French venereologist, described in 1883 the fulminant gangrene of the genitalia that bears his name, emphasizing abrupt scrotal pain and swelling in a previously healthy man, rapid progression to gangrene, and the absence of an obvious cause [44]. For most of the 20th century, survivors were left with granulating perineal and scrotal wounds managed expectantly; the reconstructive techniques developed for oncologic defects, skin grafting, scrotal advancement, thigh flaps, and later perforator flaps, were progressively applied to these post-debridement wounds so that modern management now routinely includes planned staged reconstruction [25,26]. Fournier gangrene is a synergistic necrotizing fasciitis of the perineum and genitalia with an incidence of approximately 1.6 per 100,000 men in the United States and a reported mortality that can exceed 20%, and it produces defects that differ from oncologic wounds: The field is septic rather than irradiated, debridement rather than resection defines the geometry, and the testes, penis, and anal verge are frequently exposed [24-26]. The pillars of management are early aggressive debridement, resuscitation, broad-spectrum antibiotics, and staged reconstruction once the wound is clean [25].

The reconstructive spectrum is correspondingly broad. In a systematic review of 576 reconstructions, 77.6% were achieved with comparatively simple means (direct closure, secondary healing, skin grafts, and local random flaps), with locoregional axial and perforator flaps reserved for the remaining fifth and free flaps essentially unreported [24]. Classic series established the workhorses: scrotal advancement flaps and meshed split-thickness grafts for scrotal resurfacing, superomedial thigh and pudendal thigh flaps for larger genital and perineal defects, and gracilis or ALT flaps where bulk or distant tissue is required [26,39,40]. Algorithmic approaches based on defect site and extent achieve healing with low major complication rates even in extensive disease [39,40]. Two functional lessons deserve emphasis. First, dead space behaves in sepsis as it does in oncology: gracilis muscle interposition into the perianal dead space after debridement significantly shortened the interval from reconstruction to discharge (5.1 versus 12.7 days) in a comparative series [41]. Second, reconstruction pays dividends beyond wound closure: After pedicled ALT reconstruction for Fournier defects, quality of life scores exceeded 70 of 100 in four of five domains, social functioning and self-image were restored for most survivors, and sexual health outcomes were favorable, although the disease itself durably affects sexual function and sexual health remains the most affected domain [42]. Perforator options continue to be added to this armamentarium, including the DEPAP flap for single-stage perineal coverage in compromised hosts [36].

Functional outcomes, quality of life, and sexual health

As perioperative outcomes have improved, attention has shifted to what reconstruction achieves for the patient in the long term. After extended abdominoperineal excision with gluteus maximus reconstruction, objective physical performance is largely preserved, but sitting is the persistent deficit: reduced sitting tolerance, cushion dependence, and residual perineal pain affect a substantial minority [30]. Long-term follow-up after VRAM and gluteal myocutaneous reconstruction for advanced pelvic cancer likewise documents substantial sexual dysfunction, alongside generally acceptable global quality of life, and argues for systematic preoperative counseling and structured follow-up of sexual health [37]. After lotus petal reconstruction of extralevator defects, overall quality of life is comparable to that of patients closed primarily despite larger resections, but sexual activity falls sharply (33% postoperatively versus 87% before surgery), and hip range of motion is measurably reduced [43]. In the BIOPEX-2 cohort, the presence of a perineal complication, rather than the closure technique itself, was the factor associated with lower EQ-5D and EQ-VAS scores over time [16]. Uniform, prospective measurement with validated instruments remains the exception across the field, and the heterogeneity of outcome reporting is a recurring limitation of the literature [21,22,38].

An algorithmic approach and future directions

Synthesizing the evidence, a defect-oriented algorithm can be outlined (Figure 1). For limited perineal defects without radiotherapy and with preserved levators, primary layered closure is reasonable. For irradiated abdominoperineal defects of moderate size, options include the gluteal turnover flap or other gluteal and perforator-based local flaps when the perineal phase is prone, with biologic mesh reserved for selected pelvic floor reconstructions in which flap options are unavailable. For large or exenterative defects, particularly with vaginal resection, an abdominally based VRAM (or muscle-sparing or DIEP variant) is the reference reconstruction when laparotomy and donor site permit; when the abdomen is unavailable, composite ALT vastus lateralis, gracilis, or bilateral perforator flaps are the principal alternatives. For vulvar and vulvoperineal defects, thin fasciocutaneous perforator flaps (lotus petal and pudendal territory flaps) are preferred for like-with-like resurfacing, escalating to DIEP or ALT flaps for radical defects. In Fournier gangrene, reconstruction is staged after source control and matched to the defect, from grafts and scrotal advancement to thigh and perforator flaps, with muscle interposition for perianal dead space [2,14,24,25,29].

**Figure 1:**
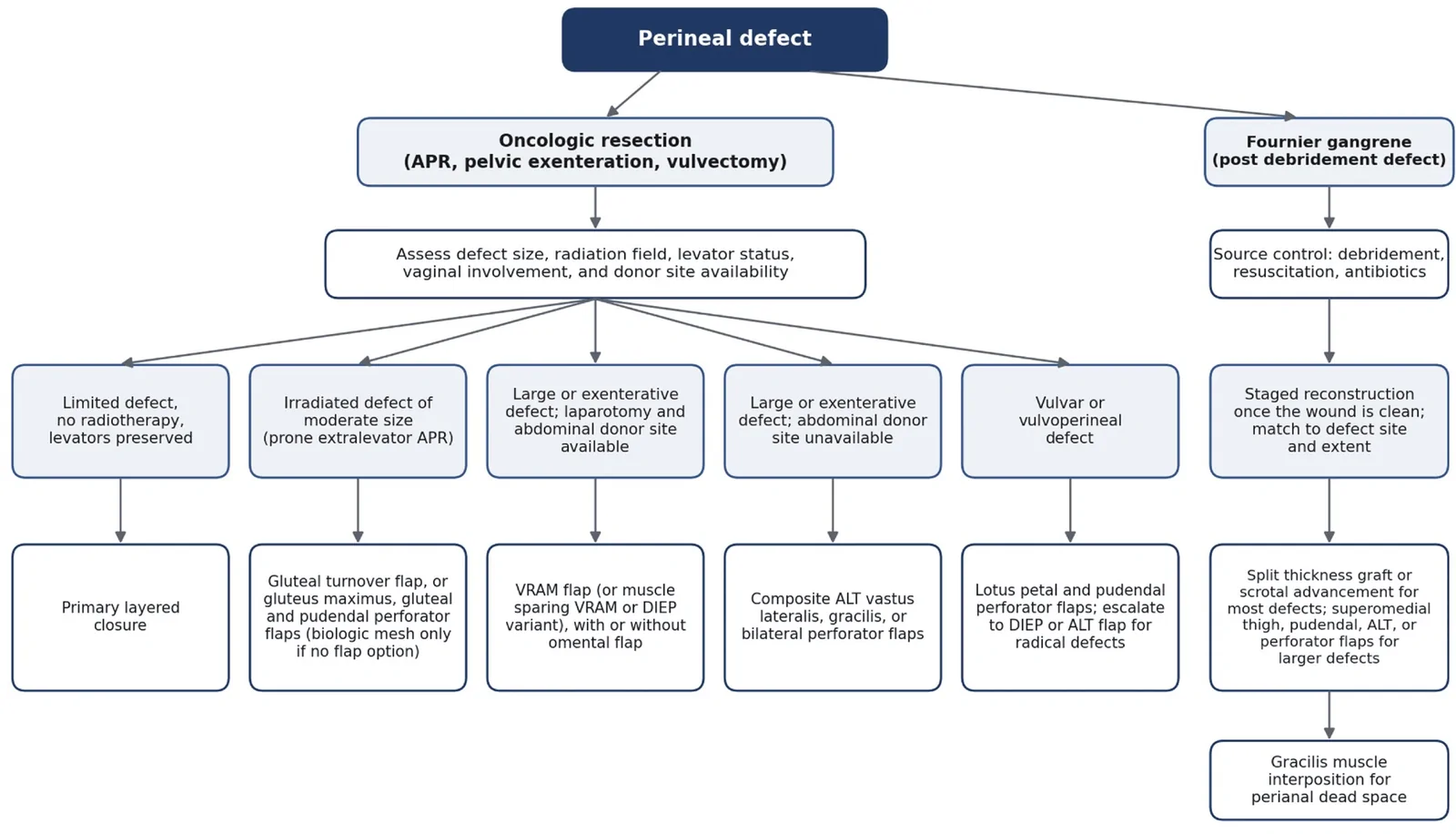
Defect-oriented algorithm for perineal reconstruction. APR, abdominoperineal resection; VRAM, vertical rectus abdominis myocutaneous; DIEP, deep inferior epigastric perforator; ALT, anterolateral thigh

Future directions include properly powered randomized comparisons among flap classes rather than against primary closure alone, standardized outcome sets incorporating sitting function and sexual health, cost-effectiveness analyses of VRAM, thigh-based and perforator reconstruction and biologic mesh beyond the first postoperative year, the wider evaluation of muscle-sparing and perforator techniques in the irradiated pelvis, and the clarification of the roles of biologic and synthetic reinforcement in perineal hernia prevention [16-18,22].

## Conclusions

Perineal reconstruction has evolved over a century from the packing of open wounds to a mature, evidence-informed discipline. The organizing principles are constant: replace what was removed with well-vascularized tissue, obliterate dead space, respect the radiation field, and plan the reconstruction with the resection rather than after it. The VRAM flap remains the standard for the largest irradiated pelvic defects; gluteal and thigh-based flaps serve the prone, extralevator excision and the abdomen-spared patient; and perforator flaps have brought donor site economy to vulvoperineal and Fournier reconstruction. Randomized evidence has begun to discipline practice, showing that not every defect needs a flap, that a simple local flap can be cost-saving even when it does not change early healing rates, and that biologic substitutes cannot yet replace vascularized tissue in the extended excision. The next advances will come less from new flaps than from better selection, standardized outcomes, economic evaluation, and multidisciplinary planning centered on the function and quality of life of the survivor.

## References

[1] Shahzad F, Ray E (2024). Pelvic and perineal reconstruction. Plast Reconstr Surg.

[2] Westbom CM, Talbot SG (2019). An algorithmic approach to perineal reconstruction. Plast Reconstr Surg Glob Open.

[3] Musters GD, Buskens CJ, Bemelman WA, Tanis PJ (2014). Perineal wound healing after abdominoperineal resection for rectal cancer: a systematic review and meta-analysis. Dis Colon Rectum.

[4] Miles WE (1908). A method of performing abdomino-perineal excision for carcinoma of the rectum and of the terminal portion of the pelvic colon. Lancet.

[5] Orticochea M (1972). The musculo-cutaneous flap method: an immediate and heroic substitute for the method of delay. Br J Plast Surg.

[6] McCraw JB, Massey FM, Shanklin KD, Horton CE (1976). Vaginal reconstruction with gracilis myocutaneous flaps. Plast Reconstr Surg.

[7] Copeland-Halperin LR, Stewart T, Chen Y, Funderburk CD, Freed GL (2020). Perineal reconstruction following abdominoperineal resection: comprehensive review of the literature. J Plast Reconstr Aesthet Surg.

[8] Nelson RA, Butler CE (2009). Surgical outcomes of VRAM versus thigh flaps for immediate reconstruction of pelvic and perineal cancer resection defects. Plast Reconstr Surg.

[9] Koshima I, Soeda S (1989). Inferior epigastric artery skin flaps without rectus abdominis muscle. Br J Plast Surg.

[10] Koshima I, Moriguchi T, Soeda S, Tanaka H, Umeda N (1992). Free thin paraumbilical perforator-based flaps. Ann Plast Surg.

[11] Allen RJ, Treece P (1994). Deep inferior epigastric perforator flap for breast reconstruction. Ann Plast Surg.

[12] Wee JT, Joseph VT (1989). A new technique of vaginal reconstruction using neurovascular pudendal-thigh flaps: a preliminary report. Plast Reconstr Surg.

[13] Yii NW, Niranjan NS (1996). Lotus petal flaps in vulvo-vaginal reconstruction. Br J Plast Surg.

[14] Sinna R, Qassemyar Q, Benhaim T, Lauzanne P, Sabbagh C, Regimbeau JM, Mauvais F (2010). Perforator flaps: a new option in perineal reconstruction. J Plast Reconstr Aesthet Surg.

[15] Holm T, Ljung A, Häggmark T, Jurell G, Lagergren J (2007). Extended abdominoperineal resection with gluteus maximus flap reconstruction of the pelvic floor for rectal cancer. Br J Surg.

[16] van den Berg R, Musters GD, Verhoef C, Blommestein HM, Tanis PJ (2026). Cost-effectiveness of the gluteal turnover flap for perineal wound closure after abdominoperineal resection: randomized clinical trial. BJS Open.

[17] Kreisel SI, Sharabiany S, Tuynman J (2025). Perineal wound closure using gluteal turnover flap after abdominoperineal resection for rectal cancer: the BIOPEX-2 randomized clinical trial. JAMA Surg.

[18] Rutegård M, Svensson J, Rutegård J (2026). Collagen implant versus gluteus maximus flap for perineal closure after extended abdominoperineal excision: NEAPE randomized clinical trial. BJS Open.

[19] Temperley HC, Shokuhi P, O'Sullivan NJ (2024). Primary closure versus vertical rectus abdominis myocutaneous (VRAM) flap closure of perineal wound following abdominoperineal resection-a systematic review and meta-analysis. Ir J Med Sci.

[20] Eseme EA, Scampa M, Viscardi JA, Ebai M, Kalbermatten DF, Oranges CM (2022). Surgical outcomes of VRAM vs. gracilis flaps in vulvo-perineal reconstruction following oncologic resection: a proportional meta-analysis. Cancers (Basel).

[21] Hellinga J, Rots M, Werker PM, Stenekes MW (2021). Lotus petal flap and vertical rectus abdominis myocutaneous flap in vulvoperineal reconstruction: a systematic review of differences in complications. J Plast Surg Hand Surg.

[22] Wendelspiess S, Kouba L, Stoffel J (2024). Perforator versus non-perforator flap-based vulvoperineal reconstruction-a systematic review and meta-analysis. Cancers (Basel).

[23] Buscail E, Canivet C, Shourick J (2021). Perineal wound closure following abdominoperineal resection and pelvic exenteration for cancer: a systematic review and meta-analysis. Cancers (Basel).

[24] Susini P, Marcaccini G, Efica J (2024). Fournier’s gangrene surgical reconstruction: a systematic review. J Clin Med.

[25] Huayllani MT, Cheema AS, McGuire MJ, Janis JE (2022). Practical review of the current management of Fournier’s gangrene. Plast Reconstr Surg Glob Open.

[26] Ferreira PC, Reis JC, Amarante JM, Silva ÁC, Pinho CJ, Oliveira IC, da Silva PN (2007). Fournier's gangrene: a review of 43 reconstructive cases. Plast Reconstr Surg.

[27] Asaad M, Mitchell D, Slovacek C (2024). Surgical outcomes of vertical rectus abdominis myocutaneous flap pelvic reconstruction. Plast Reconstr Surg.

[28] Gala Z, Lemdani MS, Ewing JN (2025). Longitudinal outcomes of abdominoperineal resection reconstruction: a single-center retrospective review. Plast Reconstr Surg Glob Open.

[29] Negosanti L, Sgarzani R, Fabbri E (2015). Vulvar reconstruction by perforator flaps: algorithm for flap choice based on the topography of the defect. Int J Gynecol Cancer.

[30] Haapamäki MM, Pihlgren V, Lundberg O, Sandzén B, Rutegård J (2011). Physical performance and quality of life after extended abdominoperineal excision of rectum and reconstruction of the pelvic floor with gluteus maximus flap. Dis Colon Rectum.

[31] Bolmstrand B, Sommar P, Nilsson PJ (2022). Vaginal reconstruction using a gluteal transposition flap after abdominoperineal excision for anorectal malignancy. Updates Surg.

[32] Rossi SA, Martineau JJ, Guillier D, Hübner M, Hahnloser D, Raffoul W, di Summa PG (2022). Outcomes of the composite anterolateral thigh flap for perineal reconstruction after postoncological abdominoperineal resection. Dis Colon Rectum.

[33] Zeng A, Qiao Q, Zhao R, Song K, Long X (2011). Anterolateral thigh flap-based reconstruction for oncologic vulvar defects. Plast Reconstr Surg.

[34] Hellinga J, Khoe PC, Stenekes MW, Eltahir Y (2018). Complications after vulvar and perineal reconstruction with a lotus petal flap. Ann Plast Surg.

[35] Bodin F, Weitbruch D, Seigle-Murandi F, Volkmar P, Bruant-Rodier C, Rodier JF (2012). Vulvar reconstruction by a "supra-fascial" lotus petal flap after surgery for malignancies. Gynecol Oncol.

[36] Kim DG, Lee KA (2025). The usefulness of DEPAP flaps for reconstructing perineal defects caused by Fournier’s gangrene: a case report. J Clin Med.

[37] Assi H, Persson A, Palmquist I, Öberg M, Buchwald P, Lydrup ML (2021). Sexual and functional long-term outcomes following advanced pelvic cancer and reconstruction using vertical rectus abdominis myocutaneous and gluteal myocutaneous flap. Eur J Surg Oncol.

[38] PelvEx Collaborative (2024). A review of functional and surgical outcomes of gynaecological reconstruction in the context of pelvic exenteration. Surg Oncol.

[39] Chen SY, Fu JP, Chen TM, Chen SG (2011). Reconstruction of scrotal and perineal defects in Fournier's gangrene. J Plast Reconstr Aesthet Surg.

[40] Tripodi D, Amabile MI, Gagliardi F (2021). Algorithm of rational approach to reconstruction in Fournier's disease. Open Med (Wars).

[41] Ismayilzade M, Dadaci M, Kendir MS, Ince B (2023). Gracilis muscle interposition to fill the perianal dead space may decrease hospital length of stay in Fournier’s gangrene. Ann Plast Surg.

[42] Rossi SA, de Schoulepnikoff C, Guillier D, Raffoul W, di Summa PG (2022). Quality of life and sexual health after perineal reconstruction in Fournier gangrene using pedicled anterolateral thigh flaps. Front Surg.

[43] Hellinga J, Stenekes MW, Werker PM, Janse M, Fleer J, van Etten B (2020). Quality of life, sexual functioning, and physical functioning following perineal reconstruction with the lotus petal flap. Ann Surg Oncol.

[44] Fournier JA (1988). Jean-Alfred Fournier 1832-1914. Gangrène foudroyante de la verge (overwhelming gangrene). Sem Med 1883. Dis Colon Rectum.

